# Conjugated Polymer Nanoparticles and Thin Films of Defect-Free Cyclic P3HT: Effects of Polymer Topology on the Nanostructure

**DOI:** 10.3390/molecules30122490

**Published:** 2025-06-06

**Authors:** Tomohisa Watanabe, Masatoshi Maeki, Manabu Tokeshi, Tianle Gao, Feng Li, Takuya Isono, Kenji Tajima, Toshifumi Satoh, Shin-ichiro Sato, Takuya Yamamoto

**Affiliations:** 1 Graduate School of Chemical Sciences and Engineering, Hokkaido University, Sapporo 060-8628, Hokkaido, Japan; t.watanabe.rs@gmail.com; 2 Division of Applied Chemistry, Faculty of Engineering, Hokkaido University, Sapporo 060-8628, Hokkaido, Japan; m.maeki@eng.hokudai.ac.jp (M.M.); tokeshi@eng.hokudai.ac.jp (M.T.); tianlegao@eng.hokudai.ac.jp (T.G.); feng.li@eng.hokudai.ac.jp (F.L.); isono.t@eng.hokudai.ac.jp (T.I.); ktajima@eng.hokudai.ac.jp (K.T.); satoh@eng.hokudai.ac.jp (T.S.); s-sato@eng.hokudai.ac.jp (S.-i.S.); 3 List Sustainable Digital Transformation Catalyst Collaboration Research Platform, Institute for Chemical Reaction Design and Discovery (ICReDD List-PF), Hokkaido University, Sapporo 001-0021, Hokkaido, Japan

**Keywords:** cyclic polymer, conjugated polymer nanoparticles, solvatochromism, grazing 30 incidence X-ray scattering, poly(3-hexylthiophene)

## Abstract

Conjugated polymer nanoparticles (CP NPs) attract attention as nanoscale materials used for a variety of applications. In relation to this, the internal structure of CP NPs is an important factor for their properties, and numerous investigations have been carried out to control their nanomorphology. Here, we report the formation of CP NPs from defect-free cyclic poly(3-hexylthiophene) (*c*-P3HT) using a microfluidic device, and the effect of polymer topology on their structural and solvatochromic properties was investigated. CP NPs from *c*-P3HT exhibited reduced particle sizes and hypsochromic shifts in the absorption spectrum when compared to CP NPs obtained from corresponding linear P3HT (*l*-P3HT). Furthermore, steady responses in the solvatochromism of CP NPs from *c*-P3HT were observed, while those from *l*-P3HT displayed molecular weight dependency. These *topology effects* were caused by the change in the conjugation length, solubility, and crystallinity upon cyclization. Grazing incidence X-ray scattering (GIXS) studies of spin-coated P3HT films further showed a reduced interchain order and a larger proportion of face-on molecular orientation on a substrate for *c*-P3HTs. The various distinct structures observed for *c*-P3HT indicate the use of polymer topology as a means of nanostructure regulation.

## 1. Introduction

**Figure 1:**
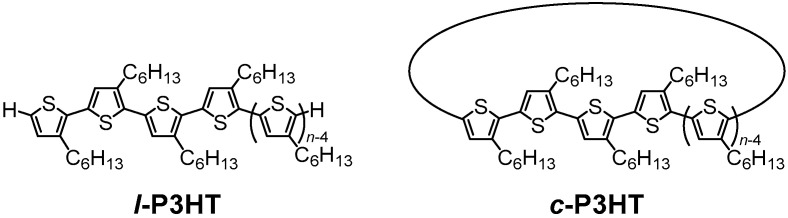
Chemical structures of *l*-P3HT and *c*-P3HT.

Cyclic polymers have a ring-like structure with a lack of chain ends. Due to this topology-induced constraint, cyclic polymers have been known to exhibit unique physical properties in both solution and bulk state [1]. For example, reduced hydrodynamic volume and melt viscosity [2,3,4], higher glass transition temperature, enhanced heat stability in self-assembled micelles [5,6], and improved crystallization speed [7,8] are some of the unique characteristics arising from the *topology effects* of cyclic polymers. Among various chemical structures of the polymer backbone, conjugated polymers have been known to exhibit unique photoelectrical properties upon cyclization. Thus, macrocyclic conjugated molecules potentially have infinite conjugation, resulting in distinct optical and electronic properties compared to their linear counterparts [9,10]. However, the actual conjugation system is often discontinued, due to twists in the polymer backbone and distortions in the ring [11]. In relation to this, we have previously reported that the synthesis and unique electronic properties of defect-free cyclic poly(3-hexylthiophene) (*c*-P3HT) shown in Figure 1, finding a fully delocalized exciton over the ring structure [12,13].

While electrooptical properties of conjugated polymers make them suitable materials for sensors and optoelectronic applications, such as active layer structure materials in optoelectronic devices [14], the preparation often involves the use of toxic organic solvents, and to achieve high crystallinity for effective charge carrier mobility, post-treatment processing such as thermal or solvent annealing is often required. A method of addressing these problems has been the fabrication and assembling of conjugated polymer nanoparticles (CP NPs), often dispersed in water [14,15,16,17,18]. Aqueous dispersions of CP NPs not only allow for an environmentally friendly method of thin-film fabrication with control over nanomorphology, but they also provide applications in biophotonic fields of CP NPs [19,20]. However, in both cases, the nanomorphology of CP NPs directly influences the physical properties of the material, indicating the need for precise control over the nanoparticle fabrication process. Various techniques are known to produce metastable dispersions of CP NPs. Among them, the nanoprecipitation method is a mild and sensitive procedure, not requiring external energy input or surfactants for nanoparticle formation, suitable for large-scale production and applications [21]. Particle formation is assumed to be due to the nucleation of small macromolecule aggregates, followed by the aggregation of these nuclei, finally ending as colloidal stability is reached [22]. Nanoparticle morphology and size are known to be influenced by various factors, such as polymer concentration, solvent/non-solvent, and rate of addition and mixing, etc. However, investigations on the use of polymer topology as a means to control nanoparticle features are rarer, but with interesting reports, where, for example, differences in stability between nanoparticles fabricated from linear and cyclic polymers have been exploited to achieve triggered drug delivery [23].

A central challenge in determining of clear effects of cyclic topology on nanoparticle properties is the difficulty in controlling the mixing process during the nanoprecipitation procedure, which inhibits precise control over the dimensions and physicochemical properties of the nanoparticles. Microfluidic platforms equipped with micromixing structures have been introduced as a means to control this mixing process, by minimizing the mixing time and providing a homogenous environment for nanoparticle growth. For example, precisely controlled NPs made from homo- and co-polymers of poly(lactic-co-glycolic) acid were fabricated using this technology, indicating potential adaptations in other hydrophobic polymers [24]. In this study, CP NPs were fabricated from defect-free linear P3HT (*l*-P3HT) and *c*-P3HT (Figure 1) with precise control using a microfluidic device equipped with a baffle-type micromixer [25,26]. *l*-P3HT and *c*-P3HT, having various degrees of polymerization, DP*_n_* (21, 26, and 43mers), are denoted as *l*-21, *l*-26, *l*-43, *c*-21, *c*-26, and *c*-43, respectively. The use of defect-free P3HT and a microfluidic device eliminated other factors that may influence the self-assembly of NPs, such as chemical structural differences and mixing conditions, thereby clarifying the topology effects of *c*-P3HT when self-assembled into NPs. The physical and optical properties of the NPs were investigated through various measurements such as dynamic light-scattering (DLS), scanning electron microscopy (SEM), and UV–Vis spectroscopy, discovering that topology effects on both the molecular level and aggregate structure level contributed to prominent differences in the size, optical, and solvatochromic properties of P3HT NPs. Moreover, the stacking and orientation of the polymer chains in thin films were studied by grazing incidence X-ray scattering (GIXS).

## 2. Results and Discussion

### 2.1. Formation of P3HT NPs

**Figure 2:**
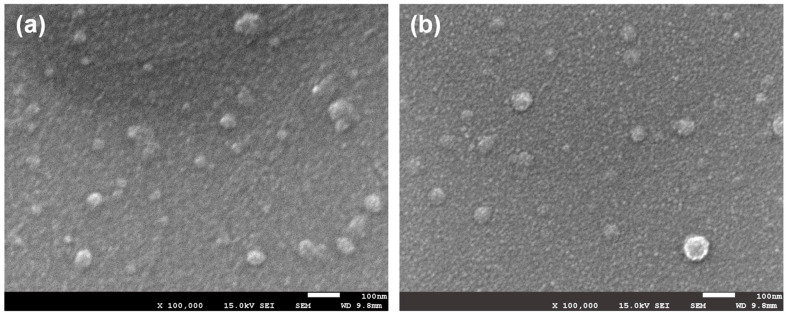
SEM images of NPs of (**a**) *l*-21 and (**b**) *c*-21. Scale bar: 100 nm.

Stable dispersions of P3HT NPs were fabricated through the nanoprecipitation method by the rapid mixing of a dilute P3HT solution in THF into water using a microfluidic device [25,26], followed by the removal of THF through heating [22]. The use of a microfluidic device enabled the automated and rapid mixture of the two solutions, therefore ensuring high reproducibility of the results. The characterization of NPs fabricated from *l*-21, *l*-26, *l*-43, *c*-21, *c*-26, and *c*-43 was carried out using DLS, ζ-potential, and SEM. The size (*z*-average) of three batches of the produced NPs determined from DLS is shown in Table 1. All samples exhibited monomodal distributions of the particle size, displaying a substantially smaller *z*-average value for *c*-P3HT NPs compared to *l*-P3HT NPs at all three DP*_n_*. For example, the *z*-average for NPs from 21mer of *c*-P3HT (*c*-21) was found to be 42 nm, which was almost half the size of those from 21mer of *l*-P3HT (*l*-21) at 82 nm. However, the correlation between particle size and DP*_n_* was not observed, producing relatively similar-sized particles for polymers of the same topology. For instance, the *z*-average value of NPs from *l*-21, *l*-26, and *l*-43 was 82, 110, and 86 nm, respectively. The correlation between the polymer topology and particle size was possibly due to the nature of the formation of NPs. According to the literature, the main forces that determine particle size and shape are the dielectric constants of the solvent and polymer concentration [22]. Since cyclic polymers exhibit reduced hydrodynamic volume in solution, the volumetric concentration of *c*-P3HT was decreased compared to *l*-P3HT, likely resulting in the reduced particle size of the *c*-P3HT NPs. The lack of any correlation between particle size and DP*_n_* of P3HT was probably due to the negligibly small volumetric concentration differences arising from the change in DP*_n_*. SEM observations of P3HT NPs (Figure 2) confirmed the formation of spherical particles with a size and size distribution consistent with the DLS data. Furthermore, the ζ-potentials of *c*-P3HT NPs were found to be around 10–20 mV higher than those of *l*-P3HT. NPs were also prepared by dropwise addition of THF solutions to water by hand, generally resulting in a significantly larger size. Thus, the NPs formed by a microfluidic device were used in the present study.

### 2.2. Optical Properties of P3HT NPs

**Figure 3:**
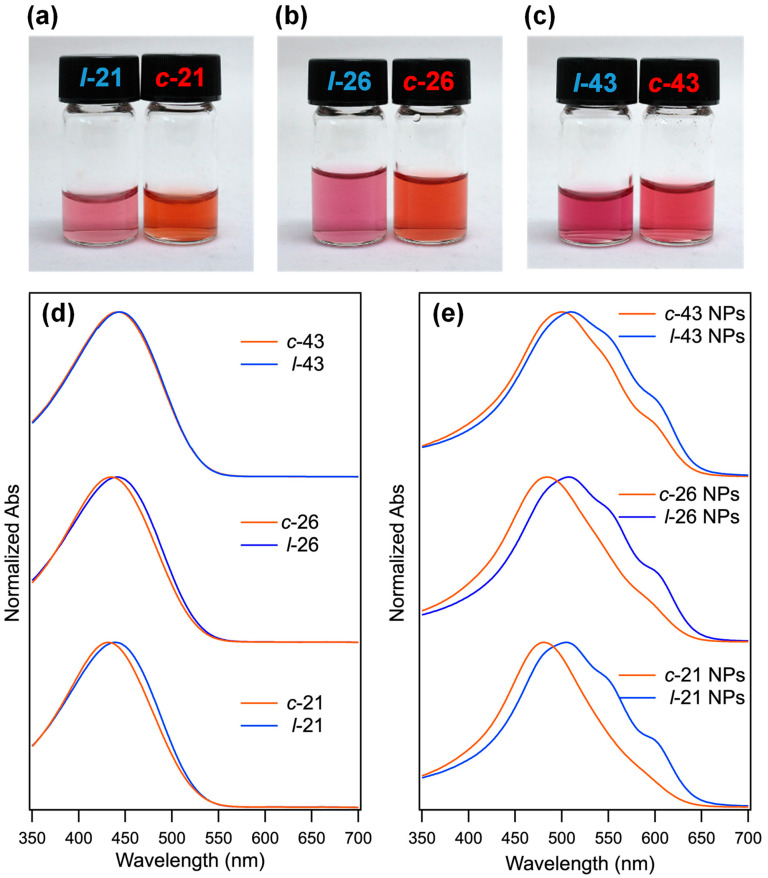
Photographs of P3HT NPs dispersions in water. (**a**) *l*-21 and *c*-21, (**b**) *l*-26 and *c*-26, and (**c**) *l*-43 and *c*-43. UV–Vis spectra of *l*-P3HT and *c*-P3HT (**d**) in THF solution and (**e**) in the NPs dispersion state in water.

In our previous report, the UV–Vis absorption spectra of molecularly dissolved *l*-P3HT and *c*-P3HT in chloroform solution were found to exhibit a decrease in conjugation length due to conformational constraints in the main chain induced upon cyclization [12,13]. Here, the absorption spectra and *λ*_max_ values of *l*-21, *l*-26, *l*-43, *c*-21, *c*-26, and *c*-43 in a THF solution and aqueous suspensions of NPs are compared in Figure 3 and Table 1. All spectra are normalized to the absorbance at *λ*_max_. The absorption spectra of P3HT in the NPs state exhibited a bathochromic shift of approximately 50 nm, compared to its solution state, with two peak shoulders arising in the wavelengths of around 550 and 600 nm, respectively, especially for *l*-P3HT NPs. Comparison between NPs fabricated from polymers of both linear and cyclic topology revealed a hypsochromic shift in *λ*_max_ of *c*-P3HT NPs against *l*-P3HT NPs at each DP*_n_*, suggesting a *topology effect* on the optical property, similar to the hypsochromic shift in the *c*-P3HT absorption spectrum in solution [12,13]. Despite the fact that the Δ*λ*_max_ between *l*-P3HT and *c*-P3HT with the corresponding DP*_n_* ranged under 10 nm in THF solutions (Δ*λ*_max, THF_ = 7, 6, and 2 nm for 21, 26, and 43mer, respectively), that in the NPs dispersion state in water was significantly enlarged to 24, 23, and 10 nm for 21, 26, and 43mer, respectively. Furthermore, the bathochromic shift of *λ*_max_ correlated with increasing DP*_n_* for both *c*-P3HT and *l*-P3HT in the NPs dispersion state as well as in solution. However, amplified Δ*λ*_max_ in the NPs dispersion state and the distinction in overall spectra shape suggest factors other than the *topology effect* on the conjugation length of P3HT to be responsible for determining *λ*_max_. The spectral shape of NPs exhibiting peak shoulders between 500 and 600 nm was similar to that of P3HT in the solid state, suggesting a bulk state-like structure within NPs. According to previous studies, the shorter wavelengths of the absorption spectrum of P3HT in bulk and NPs are attributed to the absorbance from amorphous regions, while absorbance in the longer wavelengths indicates the crystalline structure of P3HT chains [27,28]. Thus, the significantly prominent peak shoulders of *l*-P3HT NPs compared to *c*-P3HT NPs of the same DP*_n_* suggest a higher degree of crystallinity or inter-chain order within *l*-P3HT NPs. On the other hand, *c*-P3HT NPs can be considered to possess a lower degree of the inter-chain order, with the largest difference between linear and cyclic topologies observed for NPs from 21mers. From these results, it can be inferred that in the NPs dispersion state, *λ*_max_ is influenced by both the absorbance of amorphous and crystalline regions within the NPs. The smaller contribution from the crystalline absorbance in *c*-P3HT NPs leads to shorter *λ*_max_, while higher inter-chain order in *l*-P3HT NPs results in the bathochromic shift. In other words, the *topology effects* of P3HT on its NPs dispersion state were observed on two levels: one on the conjugation length of P3HT, and the other on its intermolecular aggregation behavior. Both effects contributed to the prominent differences in *λ*_max_ between *l*-P3HT and *c*-P3HT NPs.

### 2.3. Solvatochromic Properties of P3HT NPs

**Figure 4:**
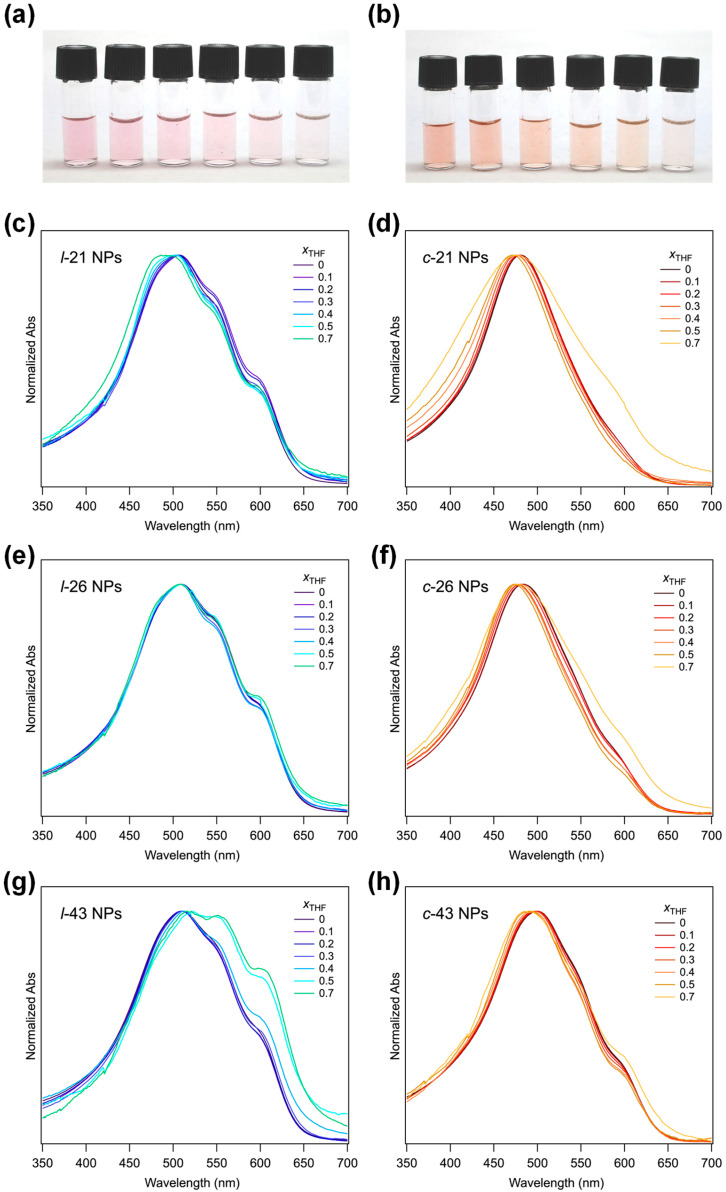
Photographs of NPs dispersions of (**a**) *l*-21 and (**b**) *c*-21 in binary mixtures of THF and water. From left to right: *x*_THF_ = 0, 0.2, 0.4, 0.6, 0.7, and 0.8. UV–Vis spectra of NPs dispersions in binary mixtures of THF and water. (**c**) *l*-21, (**d**) *c*-21, (**e**) *l*-26, (**f**) *c*-26, (**g**) *l*-43, and (**h**) *c*-43.

**Figure 5:**
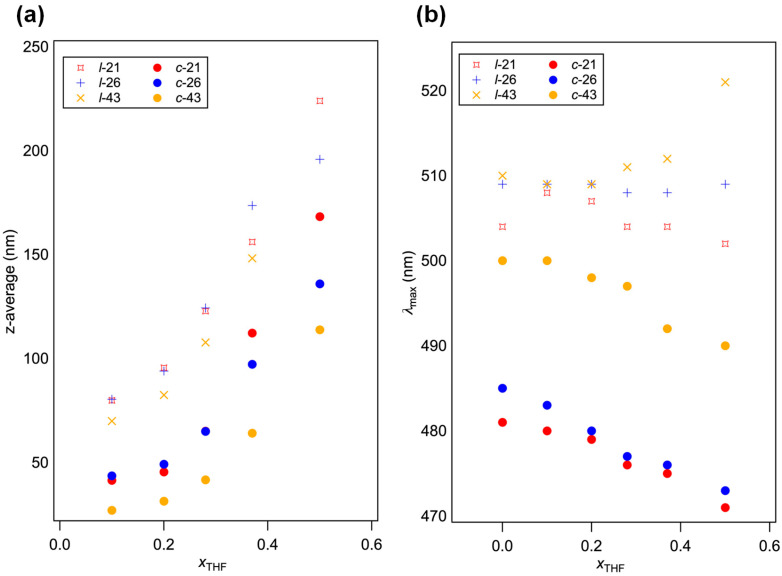
(**a**) *z*-Average and (**b**) *λ*_max_ of NPs formed from *l*-21, *c*-21, *l*-26, *c*-26, *l*-43, and *c*-43 in binary mixtures of THF and water. THF was added to the aqueous dispersions of NPs.

Self-assembled structures such as NPs and thin films of P3HT have been known to exhibit various environment-responsive characteristics. Among them, the solvatochromic properties of linear P3HT have been the target of extensive study [29]. However, topology effects on the solvatochromic properties of *c*-P3HT are yet to be addressed. Therefore, a comparative study into the solvatochromic properties of *l*-P3HT and *c*-P3HT NPs was carried out through the addition of THF into aqueous dispersions of NPs. Interestingly, distinct differences in the solvatochromic properties of *l-*P3HT and *c*-P3HT NPs were observed (Figure 4). Upon addition of THF, swelling of P3HT NPs, followed by the formation of macroscopic agglomeration of these NPs, was indicated from the exponential increase in particle size measured using DLS, as shown in Figure 5a. Refractive indices for mixtures of THF and water were set accordingly using experimental data reported by Nayak et al. [30] *λ*_max_ of THF-added P3HT NPs dispersion are shown in Figure 5b. Despite similar swelling and agglomeration behavior indicated from the DLS results between *l*-P3HT and *c*-P3HT NPs, more prominent changes in the spectra of *c*-P3HT NPs dispersions suggest contrasting responsive behaviors of the NPs according to the polymer topology, upon addition of THF. Thus, a continuous shift in *λ*_max_ was observed for *c*-P3HT NPs between THF volume ratios (*x*_THF_) of 0 and 0.5, suggesting a steady solvatochromic property. Since the dissolution of a polymer solid occurs first through swelling of amorphous regions upon contact with a good solvent, a decrease in crystallinity within *c*-P3HT NPs is deduced from the change in *λ*_max_. The steady change in *λ*_max_ of *c*-P3HT NPs could be attributed to a larger proportion of amorphous regions within NPs. In addition, *c*-P3HT exhibited better solubility in a good solvent, which may have also influenced the solvatochromic responsiveness toward the THF addition. Interestingly, upon the addition of THF to *l*-43 NPs, the overall spectrum shape changed with an increase in the absorbance around 550 and 600 nm, indicating the formation of well π-stacked structures likely by a solvent annealing effect (Figure 4g). A substantial increase in the *z*-average (from 148 nm to more than 1 μm) for *l*-43 NPs from *x*_THF_ = 0.4 to 0.5 also suggests the formation of a secondary structure of NPs.

### 2.4. Grazing Incidence X-Ray Scattering (GIXS) of P3HT Thin Films

**Figure 6:**
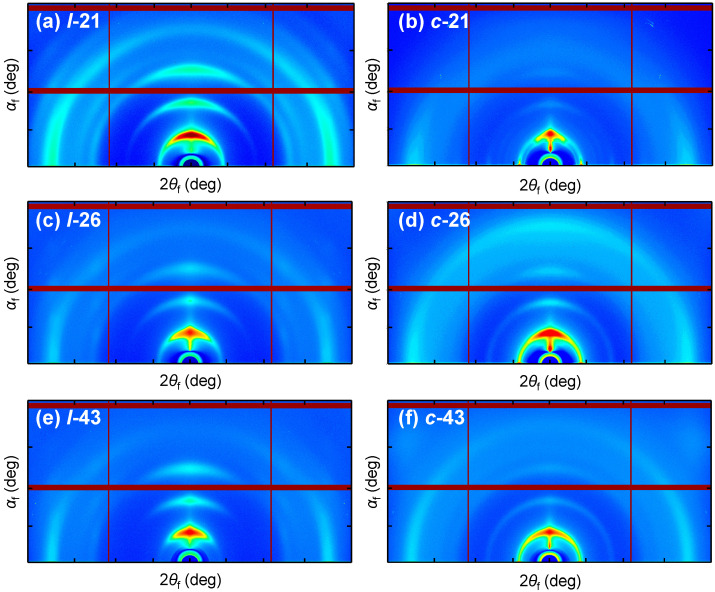
Two-dimensional grazing incidence X-ray images of *l*-P3HT and *c*-P3HT thin films spin-coated onto a silicon substrate from a THF solution. A standard heat map representation is used to visualize the scattering intensity, while the linear vertical and horizontal lines within the image correspond to spacings between the detectors, where scattering data is not obtained.

**Figure 7:**
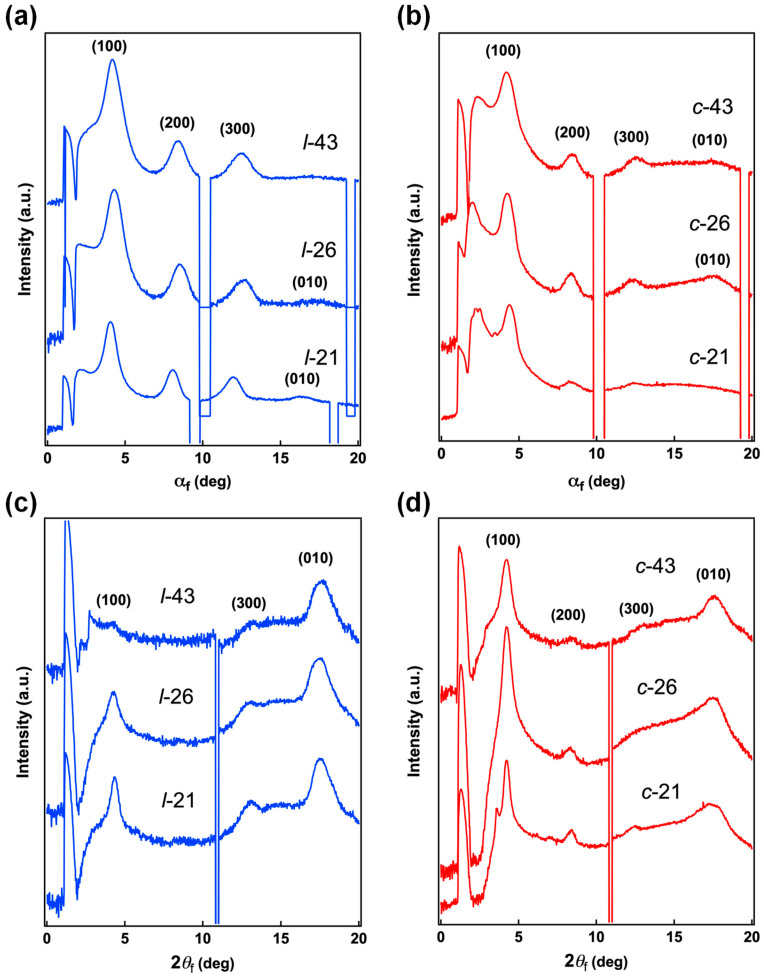
Out-of-plane and in-plane 1D data obtained from 2D grazing incidence X-ray images of *l*-P3HT and *c*-P3HT thin films spin-coated onto a silicon substrate from a THF solution. (**a**) out-of-plane, *l*-P3HT, (**b**) out-of-plane, *c*-P3HT, (**c**) in-plane, *l*-P3HT, and (**d**) in-plane *c*-P3HT.

To rationalize the effects of topology on the structural property of P3HT in the bulk state, GIXS measurements [31] were carried out for *l*-P3HT and *c*-P3HT thin films spin-coated from THF solution onto a silicon substrate. Typically, each polymer was dissolved in THF at a concentration of 0.1 wt% and spin-coated onto the substrate at a condition of 2000 rpm for 30 s. The samples were further dried overnight under reduced pressure. Two-dimensional grazing incidence X-ray images are shown in Figure 6. One-dimensional cuts of the 2D image perpendicular (out-of-plane) and parallel (in-plane) to the substrate were conducted to obtain molecular orientation information (Figure 7). Finally, the calculated d-spacing values of the scattering peaks and orientation information are summarized in Table 2.

First, *l*-21, *l*-26, and *l*-43 thin films all showed intense scattering typically seen for P3HT thin films, with strong (100) lamella scattering in the out-of-plane direction, and (010) peak from π–π stacking observed in the in-plane direction. An increase in the DP*_n_* seems to enhance the anisotropy of molecular orientation, as one can see the *l*-43 thin films exhibit the most anisotropic scattering peaks. This suggests a dominant edge-on structure within the thin film of regioregular *l*-P3HT species, in accordance with previous reports [32,33]. In contrast, the scattering peaks of *c*-P3HT thin films diminished compared to their linear counterparts. This is consistent with the results obtained from the absorbance spectra of *c*-P3HT NPs, confirming reduced crystallinity in the bulk state to be a consequence of the cyclic topology. Interestingly, in the *c*-P3HT thin films, scattering peaks from (100) appear in the in-plane direction as well as the out-of-plane direction, indicating a bimodal mixture of edge-on and face-on orientation to be dominant within the thin films of *c*-P3HT. In this case, however, the increase in the DP reduced the anisotropy, with *c*-43 displaying almost isotropic ring-like scattering, corresponding to a random molecular orientation. As shown in Table 2, calculated d-spacing values of *l*-P3HT and *c*-P3HT thin films for (100) and (010) scattering are consistent with reported values at around 1.7 nm and 0.4 nm, respectively [34]; thus, the main chain topology was found to have negligible influence on these intermolecular distances. Finally, the origins of an anomalous scattering peak (*) observed in the out-of-plane geometry of *c*-P3HT thin films are addressed. The d-spacing values were 3.28, 3.26, and 3.27 nm for *c*-21, *c*-26, and *c*-43, respectively, calculated to be approximately twice the value of the (100) scattering peaks. Whilst these peaks may correspond to the intermolecular interchain distance in the amorphous region, these signals were not as evident in the films of the linear counterparts. Moreover, their presence was only observed in the out-of-plane direction, indicating other possible origins. In any case, the present experimental *q*-range is insufficient to deduce a concrete conclusion and will be the subject of further studies.

## 3. Materials and Methods

### 3.1. Materials

*l*-21, *l*-26, *l*-43, *c*-21, *c*-26, and *c*-43 synthesized and isolated according to previous methods [12] were used. An overview of the synthesis and characterization procedures is described in the Supplementary Materials. ^1^H NMR spectra (Figure S1) and SEC traces (Figure S2) of *l*-26 and *c*-26 are shown. The following chemicals were used as received: THF super dehydrated, stabilizer-free (99.5%, Wako Pure Chemical Industries, Ltd., Osaka, Japan) for dissolution of P3HT, and Milli-Q water (Merck, Darmstadt, Germany).

### 3.2. Formation of P3HT NPs

P3HT was dissolved in THF with a polymer concentration of 0.5 mg/mL. The solutions were sonicated for 15 min and left at room temperature for over 1 h to ensure complete dissolution. Samples with precipitation were heated until a clear solution was obtained. A syringe pump was used to inject the P3HT solution and water simultaneously at the flow rate of 20 and 380 μL min^−1^, respectively, into a microfluidic device equipped with a baffle micromixer at room temperature [25,26]. THF was removed from the mixture by heating the solution on a hotplate stirrer at 85 °C for over 1 h. The NPs solutions were filtered through a 0.45-μm Toyo Roshi ADVANTEC disposable membrane filter (Toyo Roshi Kaisha, Ltd., Tokyo, Japan) unit before characterization.

### 3.3. Swelling of P3HT NPs

The swelling and redissolution of the P3HT NPs were carried out by adding arbitrary amounts of THF to the aqueous dispersions of P3HT NPs. The mixtures were then sonicated for 15 min and left still overnight at room temperature. Each sample was also shaken thoroughly by hand immediately prior to measurement.

### 3.4. DLS

DLS measurements of P3HT NPs dispersions were performed on a Malvern Panalytical Zetasizer nano S (Spectris plc, London, UK) with a 532 nm laser in a Hellma Analytics 12 μL high precision quartz cell. Measurement of each sample was repeated 3 times, where a single measurement averaged the values obtained from over 10 acquisitions. Autocorrelation curves were fitted using the cumulant fitting parameters in the Zetasizer software Ver 7.12, to obtain intensity-weighted mean hydrodynamic size, referred to herein as *z*-average size. The preset refractive index value of water was used for aqueous dispersions of P3HT NPs, while experimental values from the works of Nayak et al. were used for samples with mixed solvent composition [30].

### 3.5. ζ-Potential

ζ-Potential measurements of P3HT NPs dispersions were performed on a Malvern Panalytical Zetasizer nano S (Spectris plc, London, UK) equipped with a 633 nm laser using a Zetasizer folded capillary cell (DTS1060). Measurement of each sample was repeated 3 times, in which a single measurement averaged the values obtained from over 10 acquisitions.

### 3.6. SEM

SEM images were recorded on a JSM-7001FA instrument (JEOL, Tokyo, Japan). The accelerating voltage was 30 kV. Samples were prepared by depositing a drop of the NPs dispersion onto a silicon wafer (Nissin EM, Tokyo, Japan). An excess solution was wicked away using filter paper. The specimens were then allowed to dry under reduced pressure at room temperature. A carbon film of 10 nm thickness was vapor deposited onto each sample before observation.

### 3.7. UV–Vis Spectroscopy

The absorption spectrum of each sample was measured using a Jasco UV–Vis spectrometer V-670 (JASCO Corporation, Tokyo, Japan) in a Sansyo micro quartz cell at room temperature. Baseline measurements were carried out using the same dispersion medium, immediately before the measurements of each sample. The absorption spectra were normalized at *λ*_max_.

### 3.8. Thin Film Preparation for GIXS

Thin films of *l*-21, *l*-26, *l*-43, *c*-21, *c*-26, and *c*-43 were prepared by dissolving each sample in anhydrous stabilizer-free tetrahydrofuran (THF). In total, 60–80 μL of each solution (0.5 wt%) was spin-coated at 2000 rpm onto a silicon substrate, which was pre-cleaned by successive solvent displacement (sonication in CHCl_3_, heating in acetone at 50 °C, immersion in methanol at room temperature, and drying under reduced pressure).

### 3.9. GIXS Measurements

GIXS measurements for thin film samples were performed at the 3C beamline of the Pohang Accelerator Laboratory (PAL; Pohang, Republic of Korea). Scattering data were collected at room temperature using X-ray radiation sources with a wavelength of 1.21 Å and an Eiger X4M detector (Dectris, Baden, Switzerland). The exposure time was set to 40–60 s. The sample-to-detector distance was calibrated using silver behenate as a standard sample.

## 4. Conclusions

We have shown that the *topology effects* of *c*-P3HT can be utilized to control both size and structure within NPs, resulting in reduced size and smaller *λ*_max_. Since *c*-P3HT NPs tended to have a smaller proportion of crystalline regions and shorter conjugation lengths, *λ*_max_ lay in relatively short wavelengths. On the other hand, the extended conjugation length and a larger proportion of crystalline regions in *l*-P3HT NPs contributed to longer *λ*_max_. The solvatochromic properties of P3HT NPs were also topology-dependent. Upon the addition of various amounts of THF to aqueous dispersions of P3HT NPs, a steady change in the absorption spectrum was observed for *c*-P3HT NPs, while *l*-P3HT NPs displayed inconsistent transitions. The lower degree of crystallinity and increased solubility in THF of *c*-P3HT originating from the reduced hydrodynamic volume can be regarded as *topology effects* on the properties of *c*-P3HT NPs. This work demonstrates an alternative methodology of controlling the structural aspects of CP NPs, which in turn amplifies the optical difference between *l*-P3HT and *c*-P3HT NPs.

GIXS results showed that from similar d-spacing values of (100) and π-stacking peaks between *l*-P3HT and *c*-P3HT thin films, molecular topology does not significantly influence the intermolecular distance. Moreover, from the 2D scattering images, the molecular orientation within the thin films clearly differed between *l*-P3HT and *c*-P3HT; *l*-P3HT produced clear “edge-on” traits, which became increasingly significant in accord with the increase in DP*_n_*. Contrastingly, *c*-P3HT produced a mixture of “edge-on” and “face-on” orientation, and this trend seemed to diminish upon the increase in DP*_n_*.

## Figures and Tables

**Table 1:** Properties of *l*-P3HT and *c*-P3HT in THF solution and in the NPs dispersion state in water.

		*l*-21 *^c^*	*c*-21 *^c^*	*l*-26 *^c^*	*c*-26 *^c^*	*l*-43 *^c^*	*c*-43 *^c^*
*M*_n,SEC_ (kg mol^−1^)		3.5	2.9	4.3	3.1	7.2	5.7
*M*_p,SEC_ (kg mol^−1^)		3.5	2.9	4.1	3.1	8.7	6.2
*M*_w_/*M*_n_		1.05	1.04	1.08	1.05	1.13	1.19
*z*-average size (nm) *^a^*		82 ± 6	42 ± 5	110 ± 20	55 ± 3	86 ± 9	33 ± 1
ζ-potential (mV) *^b^*		–56 ± 2	–45 ± 1	–54 ± 1	–34 ± 4	–64 ± 3	–42 ± 5
in the NPs dispersion state in water	*λ*_max, water_ (nm)	505	481	507	484	510	500
	Δ*λ*_max, water_ (nm)	24		23		10	
in THF solution	*λ*_max, THF_ (nm)	439	432	441	435	444	442
	Δ*λ*_max, THF_ (nm)	7		6		2	

*^a^* Average of 3 independent samples precipitated from the same polymer solution in THF determined from DLS measurements. *^b^* Average of 5 consecutive measurements of a single sample. *^c^* “*l*-21” and “*c*-21” indicate 21mer of *l*-P3HT and *c*-P3HT, respectively. The same shall apply to the other DP*_n_*.

**Table 2:** d-Spacing and the molecular orientation of *l*-P3HT and *c*-P3HT in thin films.

			*l*-21	*c*-21	*l*-26	*c*-26	*l*-43	*c*-43
d-spacing [nm]	(100) lamella	Vertical	1.80	1.67	1.71	1.72	1.76	1.76
		Horizontal	1.63	1.68	1.66	1.68	1.70	1.67
	(010) π–π stacking	Vertical	0.44	-	0.42	0.41	-	0.41
		Horizontal	0.41	0.41	0.41	0.41	0.40	0.40
Molecular Orientation			edge-on	bimodal (edge-on/face-on mixture)	edge-on	bimodal (edge-on/face-on mixture)	edge-on	random

## Data Availability

The raw data supporting the conclusions of this article will be made available by the authors on request.

## Supplementary Materials

The following supplementary materials can be downloaded at https://www.mdpi.com/article/10.3390/molecules30122490/s1, Synthetic procedures of linear and cyclic P3HT; Determination of molecular weights from size-exclusion chromatography (SEC); Figure S1: ^1^H NMR spectra of *l*-26 and *c*-26; Figure S2: SEC traces of *l*-26 and *c*-26.
