# Supplementary material for: Conjugated Polymer Nanoparticles and Thin Films of Defect-Free Cyclic P3HT: Effects of Polymer Topology on the Nanostructure
Source: Molecules. 2025 Jun 6;30(12):2490. doi: 10.3390/molecules30122490 (PMC12196461; doi:10.3390/molecules30122490)
Supplement: Supplementary file 1 [file molecules-30-02490-s001.zip › molecules-3634183-supplementary.pdf]

Supplementary Materials

Conjugated Polymer Nanoparticles and Thin Films of Defect-Free Cyclic P3HT: Effects of Polymer Topology on the Nanostructure

Tomohisa Watanabe [1], Masatoshi Maeki [2], Manabu Tokeshi [2], Tianle Gao [2], Feng Li [2], Takuya Isono [2], Kenji Tajima [2], Toshifumi Satoh [2,3], Shin-ichiro Sato [2], and Takuya Yamamoto [2,*]

1 Graduate School of Chemical Sciences and Engineering, Hokkaido University, Sapporo, Hokkaido 060–8628, Japan

2 Division of Applied Chemistry, Faculty of Engineering, Hokkaido University, Sapporo, Hokkaido, 060-8628, Japan

3 List Sustainable Digital Transformation Catalyst Collaboration Research Platform, Institute for Chemical Reaction Design and Discovery (ICReDD List-PF), Hokkaido University, Sapporo, Hokkaido 001-0021, Japan

* E-mail: yamamoto.t@eng.hokudai.ac.jp.

1. Synthetic procedures of linear and cyclic P3HT

The polymers used in this study were synthesized according to our previous reports.[1, 2] First, synthesis of linear P3HT (***l*-P3HT**) was conducted through a Grignard metathesis (GRIM) polymerization using an initiator. The subsequent synthesis of defect-free cyclic P3HT (*c*-P3HT) was achieved by introducing a trimethylstannyl group at both ends of linear P3HT, followed by an intramolecular cyclization reaction. Side products including *l*-P3HT with non-regioregular structure and non-cyclized species were removed by a scavenging method, where terminal groups of the *l*-P3HT were reacted with surface functionalized resins. A final preparative size exclusion chromatography (SEC) (Japan Analytical Industry LC-9201 recycling preparative HPLC system equipped with JAIGEL-2H and 3H columns. Eluent: $CHCl_3$. Flow rate: 3.5 mL min^{-1}) was used to fractionate *c*-P3HT. ^{1}H NMR spectra and SEC traces of *l*-26 and *c*-26 are shown in Figures S1 and S2, respectively.

2. Determination of molecular weights from size-exclusion chromatography (SEC)

SEC measurements were conducted on a Shodex GPC-101 gel permeation chromatography system (Shodex DU-2130 dual pump, RI-71 detector, and Shodex ERC-3125SN degasser) equipped with a Shodex KF-G guard column (4.6 mm × 10 mm; pore size, 8 μm) and two Shodex KF-804L columns (8 mm × 300 mm) in series. THF was used as an eluent (flow rate of 1.0 mL min^{-1}, at 40 °C), and polystyrene standard samples were used as calibration standards. The molecular weights of P3HT were estimated by using a correction factor where the following equation: $M_{n,SEC(P3HT)} = M_{n,SEC(PS)}/1.67$ was used, according to literature.[3]

References

1. Yamamoto, T.; Hosokawa, M.; Nakamura, M.; Sato, S.; Isono, T.; Tajima, K.; Satoh, T.; Sato, M.; Tezuka, Y.; Saeki, A.; et al. Synthesis, isolation, and properties of all head-to-tail cyclic poly(3-hexylthiophene): Fully delocalized exciton over the defect-free ring polymer. *Macromolecules* **2018**, *51*, 9284–9293.

2. Sato, R.; Utagawa, A.; Fushimi, K.; Li, F.; Isono, T.; Tajima, K.; Satoh, T.; Sato, S.; Hirata, H.; Kikkawa, Y.; et al. Molecular Weight-Dependent Oxidation and Optoelectronic Properties of Defect-Free Macrocyclic Poly(3-hexylthiophene). *Polymers* **2023**, *15*, 666.

3. Koch, F.P.V.; Rivnay, J.; Foster, S.; Muller, C.; Downing, J.M.; Buchaca-Domingo, E.; Westacott, P.; Yu, L.Y.; Yuan, M.J.; Baklar, M.; et al. The impact of molecular weight on microstructure and charge transport in semicrystalline polymer semiconductors poly(3-hexylthiophene), a model study. *Prog. Polym.* **2013**, *38*, 1978–1989.

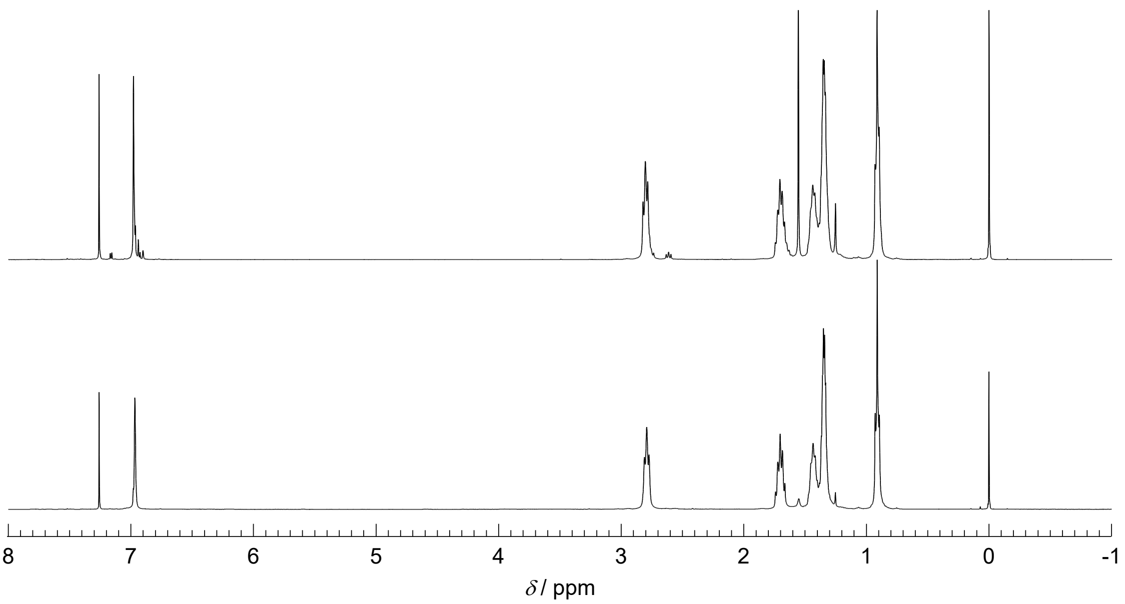

Figure S1. ^{1}H NMR spectra of *l*-26 (top) and *c*-26 (bottom).

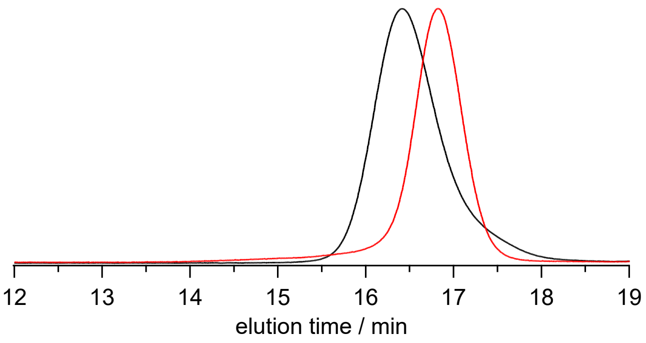

Figure S2. SEC traces of *l*-26 (black) and *c*-26 (red).
